# Impact of Social Vulnerability and Allostatic Load on Postoperative Outcomes

**DOI:** 10.1245/s10434-025-18144-5

**Published:** 2025-08-20

**Authors:** Mujtaba Khalil, Selamawit Woldesenbet, Jasmine King, Shreya Shaw, Zayed Rashid, Abdullah Altaf, Shahzaib Zindani, Samilia Obeng-Gyasi, Timothy M. Pawlik

**Affiliations:** Department of Surgery, The Ohio State University Wexner Medical Center and James Comprehensive Cancer Center, Columbus, OH USA

**Keywords:** Allostatic load, Surgical outcomes, Social vulnerability, Hepatopancreatobiliary surgery

## Abstract

**Background:**

We sought to investigate the association between allostatic load (AL), social vulnerability, and postoperative outcomes following hepatopancreatobiliary (HPB) cancer surgery.

**Methods:**

Individuals who underwent HPB surgery were identified using the Epic Cosmos database. AL was calculated based on 10 biomarkers across four physiological systems: cardiovascular, metabolic, renal, and immune. Multivariable regression models were used to examine the association between AL, postoperative outcomes, and failure to rescue (FTR).

**Results:**

Among 34,253 individuals, mean patient age was 71 years (interquartile range 63–78). Approximately half of patients were male (*n* = 18,045, 52.7%) and had a high Charlson Comorbidity Index (CCI) score (CCI >2; *n* = 29,246, 85.4%). The most common cancer site was the pancreas (*n* = 21,402, 62.5%), followed by the liver (*n* = 8451, 24.7%) and the biliary tract (*n* = 4400, 12.8%). Overall, 13.8% (*n* = 4717) of patients had high AL. On multivariable analysis, the risk of allostasis increased stepwise with higher social vulnerability (reference: low; medium: odds ratio [OR] 1.11, 95% confidence interval [CI] 1.04–1.19; high: OR 1.17, 95% CI 1.11–1.17). Moreover, high AL was associated with a 44% increased risk of Clavien–Dindo grade IV complications (OR 1.44, 95% CI 1.36–1.54) and an 85% increased risk of FTR (OR 1.85, 95% CI 1.60–2.13). In addition, the risk of 30-day mortality was approximately twofold higher with elevated AL (OR 1.92, 95% CI 1.70–2.19).

**Conclusion:**

Individuals residing in socially vulnerable areas experience socioeconomic stressors that contribute to long-term physiological damage, resulting in worse outcomes following surgery.

**Supplementary Information:**

The online version contains supplementary material available at 10.1245/s10434-025-18144-5.

Hepatopancreatobiliary (HPB) cancer incidence and mortality have steadily increased over the past few decades.^1^ Individuals from minoritized groups and socially vulnerable neighborhoods experience a higher risk of HPB cancer and poorer surgical outcomes.^2,3^ Of note, social vulnerabilities such as poverty, limited transportation, and food insecurity hinder access to care and contribute to these disparities.^3–5^ Long-term exposure to these challenges may also disrupt psychological and physiological systems, potentially affecting recovery after treatment.^6,7^

One framework for understanding the physiological impacts of social vulnerability on health is through calculating allostatic load (AL).^8^ Allostasis refers to the cumulative effects of long-term stress exposure on the human body.^8^ When stress arises, the body recalibrates its adaptive mechanisms to maintain balance.^8^ If the demands exceed an individual’s capacity to adapt, allostatic overload may occur, disrupting metabolic, immune, cardiac, and hematologic functions.^8^ McEwen and Stellar first introduced the concept of allostasis, using individual biomarkers to calculate a score that could predict wear and tear in the body.^9^ These biomarkers include anthropometric, inflammatory, metabolic, and cardiovascular measurements.^9^ AL has been recognized as a significant predictor of various chronic diseases, including cardiovascular disease, diabetes, musculoskeletal disorders, and neurological conditions.^10–12^ HPB cancer outcomes are of particular interest due to their rising incidence, largely driven by lifestyle factors and socioeconomic status, both key contributors to allostasis.^13^ Additionally, emerging evidence suggests an association between AL and conditions such as hepatic steatosis and liver fibrosis, which increase the risk of hepatocellular carcinoma.^13^

Given that previous studies have identified structural factors associated with poor outcomes, social vulnerability may lead to epigenetic changes and disruptions in biological processes, resulting in slower recovery and worse surgical outcomes.^8,11,12^ Nonetheless, the association between allostasis and surgical outcomes among patients undergoing HPB cancer surgery remains ill-defined. Therefore, the objective of the current study was to determine the association between social vulnerability, AL, and postoperative outcomes among patients with HPB cancer.

## Methods

### Data Source and Patient Selection

The Epic Cosmos database was queried using International Classification of Diseases, Tenth Edition (ICD-10) codes to obtain data on individuals who underwent HPB surgery for a malignant indication (electronic supplementary material [ESM] Table 1). Epic Cosmos is a consolidated database of electronic health records (EHRs) sourced from health systems throughout the United States.^14^ Epic Cosmos is one of the largest databases nationwide, encompassing 257 million patients and over 36,000 hospitals and clinics.^14^ A key strength of the Epic Cosmos database is its inclusion of socioeconomic data at the ZIP code level, in addition to clinical data.^15^ The study included individuals aged 18 years and older who underwent HPB surgery between 2013 and 2020 for a malignant indication. The time period between 2013 and 2020 was selected because this period represents the earliest year that all required clinical and biomarker data for AL calculation became widely available across contributing Epic Cosmos sites. Limiting the analysis to 2020 ensured consistency in data reporting and allowed for adequate follow-up while avoiding incomplete data from later years with ongoing uploads. Patients who did not receive surgery, as well as those with stage 0 or IV disease, were excluded. The study followed the Strengthening the Reporting of Observational Studies in Epidemiology (STROBE) guideline for observational studies. The Institutional Review Board at Ohio State University approved the study, and informed consent was waived because the data were de-identified.

### Allostatic Load

**Fig. 1:**
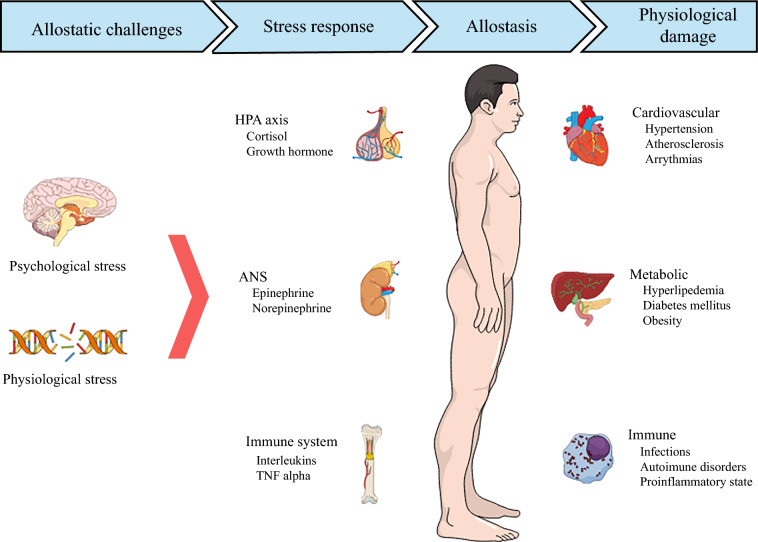
Schematic representation of allostasis and associated physiological damage. *HPA* hypothalamic-pituitary-adrenal, *ANS* Autonomic Nervous System

AL is a composite measure of physiological damage caused by cognitive and emotional stressors.^8^ Figure 1 demonstrates how socioeconomic stressors contribute to allostasis and physiological damage. AL was calculated using the methodology established by Obeng-Gyasi et al and Chen et al.^12,16^ Laboratory parameters from the year preceding HPB cancer diagnosis were analyzed, focusing on 10 biomarkers across four physiological systems.^12,16^ The systems and their respective biomarkers were (1) cardiovascular, i.e. heart rate (HR), systolic blood pressure (SBP), and diastolic blood pressure (DBP); (2) metabolic, i.e. body mass index (BMI), alkaline phosphatase (ALP), blood glucose, and albumin; (3) renal, i.e. creatinine (Cr) and blood urea nitrogen (BUN); and (4) immunologic, i.e. white blood cell (WBC) count.^12,16^ Each biomarker’s distribution was assessed, with patients receiving one point if their biomarker value fell within the worst quartile.^12,16^ For example, values at or above the 75th percentile for HR, SBP, DBP, BMI, ALP, blood glucose, WBC, Cr, and BUN were assigned one point. Additionally, individuals with albumin values at or below the 25th percentile were assigned one point. The points were summed to calculate a composite AL score ranging from 0 to 10, with higher scores indicating greater physiological damage. These scores were then categorized as high or low based on the cohort’s median AL score.

### Covariates and Outcomes of Interest

Patient-level data on age, sex, marital status, race (categorized as White, Black, Asian, or Other [American Indian, Hispanic, and Alaska Native]), Charlson Comorbidity Index (CCI), cancer site (liver, pancreas, and biliary tract), year of diagnosis, Social Vulnerability Index (SVI), and residential area (metropolitan vs. non-metropolitan) were collected. SVI is a composite measure of community vulnerability and resilience to external pressures,^17^ and is calculated based on 18 community-level factors such as poverty, education, housing, and minority status; a high SVI represents socially vulnerable patients.^17^

The primary outcomes of interest were postoperative complications and failure to rescue (FTR). Postoperative complications were defined according to the Clavien–Dindo (CD) classification. While the CD classification classifies grade III complications as ‘major’, we limited our analysis to grade IV complications due to the availability of validated algorithms that reliably identify these events in large administrative databases.^18,19^ In contrast, grade III complications are more heterogeneous and less consistently captured in structured data, which may compromise analytic accuracy and reproducibility in large datasets. CD grade IV complications included unplanned intubation, pulmonary embolism, ventilator support for more than 48 h, acute renal failure, cardiac arrest requiring cardiopulmonary resuscitation, acute myocardial infarction, and severe sepsis or septic shock.^18,19^ Acute renal failure was defined as worsening renal function requiring hemodialysis, peritoneal dialysis, hemofiltration, hemodiafiltration, or ultrafiltration in a patient who did not require dialysis before surgery.^18^ FTR was defined as in-hospital death in a patient with any of the above-mentioned complications during index admission. Extended length of stay (LOS) and discharge to home were studied as secondary outcomes.

### Statistical Analysis

Descriptive statistics were presented as medians with interquartile ranges (IQRs) for continuous variables and as frequencies and percentages for categorical variables. Differences in baseline characteristics were assessed using the Kruskal–Wallis test for continuous variables and the Chi-square or Fisher’s exact tests for categorical variables. Multivariable logistic regression models examined the association between AL and CD grade IV complications, as well as between AL and FTR. The models adjusted for age, sex, marital status, CCI, year of diagnosis, cancer site, surgical approach, and residential area. Missing values were handled using Multiple Imputation by Chained Equations (MICE). To test result robustness, analyses were conducted on both imputed and non-imputed datasets, yielding consistent outcomes. Statistical tests were two-tailed with a significance level of *p* < 0.05. Analyses were performed using SAS 9.4 (SAS Institute, Inc., Cary, NC, USA).

## Results

### Baseline Characteristics of Patients

A total of 34,253 patients underwent HPB surgery for a malignant indication. Mean age at the time of surgery was 71 years (IQR 63–78) and most patients were male (*n* = 18,045, 52.7%). Majority of patients had a high CCI score (CCI >2: *n* = 29,246, 85.4%), were married (*n* = 20,834; 61.6%), and lived in metropolitan areas (*n* = 27,781, 81.5%). Moreover, most individuals self-identified as White (*n* = 27,303, 80.3%), followed by smaller groups identifying as Black (*n* = 3940, 11.6%), Asian (*n* = 1495, 4.4%), or another race/ethnicity (*n* = 1249, 3.7%). The most common cancer site was the pancreas (*n* = 21,402, 62.5%), followed by the liver (*n* = 8451, 24.7%) and the biliary tract (*n* = 4400, 12.8%) (Table 1). Following surgery, the overall incidence of complications was 45% (*n* = 15,421), with 19.3% (*n* = 6622) of patients experiencing CD grade IV complications. Moreover, 8.5% (*n* = 2909) of patients were admitted to the intensive care unit (ICU), and the FTR rate was 2.3% (*n* = 777). In terms of discharge disposition, 55.3% (*n* = 18,937) were discharged home, and 30-day mortality was 3% (*n* = 1018) (Table 2).

**Table 1:** Baseline characteristics of patients stratified by high and low allostatic load

Characteristics	Total (*N* = 34,253)	Allostatic load		*p* Value
		Low (*n* = 29,539, 86.2%)	High (4714, 13.8%)	
Age, years	71 (63–78)	71 (63–77)	73 (65–79)	< 0.001
Sex				< 0.001
Female	16,208 (47.3)	14,494 (49.1)	1714 (36.4)	
Male	18,045 (52.7)	15,045 (50.9)	3000 (63.6)	
Marital status				0.203
Single	13,001 (38.4)	11,250 (38.6)	1751 (37.6)	
Married	20,834 (61.6)	17,926 (61.4)	2908 (62.4)	
Race				< 0.001
White	27,303 (80.3)	23,635 (80.6)	3668 (78.4)	
Black	3940 (11.6)	3207 (10.9)	733 (15.7)	
Asian	1495 (4.4)	1376 (4.7)	119 (2.5)	
Other	1249 (3.7)	1088 (3.7)	161 (3.4)	
Charlson Comorbidity Index				< 0.001
≤ 2	5007 (14.6)	4668 (15.8)	339 (7.2)	
> 2	29,246 (85.4)	24,871 (84.2)	4375 (92.8)	
Cancer site				< 0.001
Pancreas	21,402 (62.5)	18,513 (62.7)	2889 (61.3)	
Liver	8451 (24.7)	7332 (24.8)	1119 (23.7)	
Biliary tract	4400 (12.8)	3694 (12.5)	706 (15.0)	
Social Vulnerability Index				< 0.001
Low	11,599 (34.1)	10,148 (34.6)	1451 (30.9)	
Medium	11,550 (33.9)	9912 (33.8)	1638 (34.9)	
High	10,873 (32.0)	9273 (31.6)	1600 (34.1)	
Year of diagnosis				0.112
2016	1969 (5.7)	1703 (5.8)	266 (5.6)	
2017	2715 (7.9)	2349 (8.0)	366 (7.8)	
2018	3440 (10.0)	2945 (10.0)	495 (10.5)	
2019	3824 (11.2)	3285 (11.1)	539 (11.4)	
2020	3877 (11.3)	3342 (11.3)	535 (11.3)	
2021	4377 (12.8)	3754 (12.7)	623 (13.2)	
2022	4693 (13.7)	4108 (13.9)	585 (12.4)	
2023	5312 (15.5)	4545 (15.4)	767 (16.3)	
2024	4046 (11.8)	3508 (11.9)	538 (11.4)	
Residential area				
Metropolitan	27,781 (81.5)	24,031 (81.7)	3750 (79.7)	
Non-metropolitan	6327 (18.5)	5373 (18.3)	954 (20.3)

**Table 2:** Postoperative outcomes stratified by high and low allostatic load

Characteristics	Total (*N* = 34,253)	Allostatic load		*p* Value
		Low (*n* = 29,539, 86.2%)	High (4714, 13.8%)	
Complications	15,421 (45.0)	12,854 (43.5)	2567 (54.5)	< 0.001
Myocardial infarction	2599 (7.6)	2073 (7.0)	526 (11.2)	< 0.001
Venous thromboembolism	2549 (7.4)	2099 (7.1)	450 (9.5)	< 0.001
Surgical site infection	9057 (26.4)	7669 (26.0)	1388 (29.4)	< 0.001
Respiratory failure	1523 (4.4)	1051 (3.6)	472 (10.0)	< 0.001
Pneumonia	2163 (6.3)	1792 (6.1)	371 (7.9)	< 0.001
Hemorrhage	2039 (6.0)	1696 (5.7)	343 (7.3)	< 0.001
Clavien–Dindo grade IV complications	6622 (19.3)	5381 (18.2)	1241 (26.3)	< 0.001
ICU admission	2909 (8.5)	2420 (8.2)	489 (10.4)	< 0.001
Failure to rescue	777 (2.3%)	582 (2.0)	195 (4.1)	< 0.001
Extended length of stay	7806 (22.8)	6458 (21.9)	1348 (28.6)	< 0.001
Discharge to home	18,937 (55.3)	16,721 (56.6)	2216 (47.0)	< 0.001
30-day mortality	1018 (3.0)	753 (2.5)	265 (5.6)	< 0.001

*ICU* intensive care unit

### Risk Factors for High Allostatic Load

Median AL was 6 (IQR 4–7) and 13.8% (*n* = 4717) of patients had high AL. Of note, males (high AL: 63.6% vs. low AL: 50.9%), older individuals (high AL: 73 years [IQR 65–79] vs. low AL: 71 years [IQR 63–77]), and patients with a high CCI (CCI >2: high AL: 92.8% vs. low AL: 84.2%) were more likely to have high AL (all *p* < 0.001). Similarly, Black patients (high AL: 15.7% vs. low AL: 10.9%) and individuals living in medium (high AL: 34.9% vs. low AL: 33.8%) or high SVI neighborhoods (high AL: 34.1% vs. low AL: 31.6%) were also more likely to have high AL (all *p* < 0.001) (Table 2). On multivariable analysis, older age (odds ratio [OR] 1.01, 95% confidence interval [CI] 1.01–1.0), male sex (OR 1.70, 95% CI 1.60–1.80), a high CCI score (OR 2.21, 95% CI 2.02–3.44), and residence in a non-metropolitan area (OR 1.09, 95% CI 1.02–1.17) were independently associated with high AL. Notably, the risk of allostasis increased stepwise with higher SVI (reference: low; medium: OR 1.11, 95% CI 1.04–1.19; high: OR 1.17, 95% CI 1.11–1.17) (ESM Table 2).

### Allostatic Load and Postoperative Outcomes

**Fig. 2:**
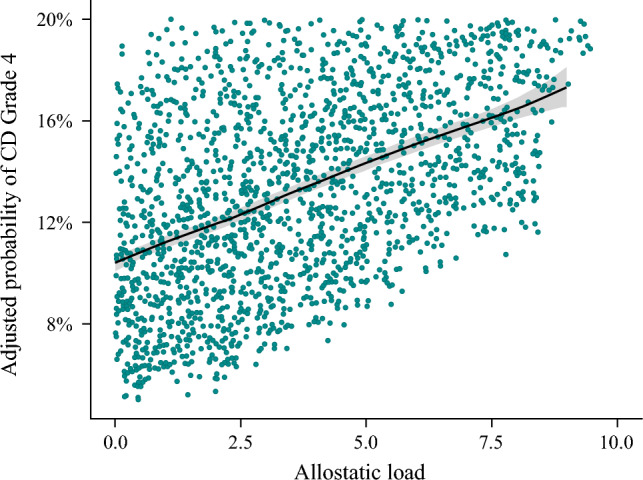
Adjusted risk of CD grade IV complications, stratified by allostatic load score. *CD* Clavien–Dindo

Patients with high AL were more likely to experience CD grade IV complications (high AL: 26.3% vs. low AL: 18.2%) and ICU admission (high AL: 10.4% vs. low AL: 8.2%) [both *p* < 0.001]. Additionally, individuals with high AL had extended LOS (high AL: 28.6% vs. low AL: 21.9%) and were more likely to experience FTR (high AL: 4.1% vs. low AL: 2.0%) [both *p* < 0.001]. In terms of discharge disposition, patients with high AL were less likely to be discharged home (high AL: 47.0% vs. low AL: 56.6%) and more likely to die within 30 days (high AL: 5.6% vs. low AL: 2.5%) [both *p* < 0.001] (Table 2). On multivariable analysis, high AL was associated with a 44% increased risk of CD grade IV complications (OR 1.44, 95% CI 1.36–1.54) and an 85% increased risk of FTR (OR 1.85, 95% CI 1.60–2.13). Notably, the risk of CD grade IV complications increased with higher AL (Fig. 2). Moreover, the risk of 30-day mortality was approximately twofold higher with elevated AL (OR 1.92, 95% CI 1.70–2.19) (Table 3). These associations remained consistent in subanalyses stratified by CCI, race, and cancer site.

**Table 3:** Multivariable logistic regression examining the association between high allostatic load and postoperative outcomes (reference: low allostatic load)

Outcomes	OR	95% CI	*p *Value
Clavien–Dindo grade IV complications	1.44	1.36–1.54	< 0.001
Failure to rescue	1.85	1.60–2.13	< 0.001
Discharge to home	0.71	0.67–0.75	< 0.001
30-day mortality	1.92	1.70–2.19	< 0.001

*OR* odds ratio, *CI* confidence interval

## Discussion

The graded relationship between social vulnerability and health reflects a dynamic interplay of several mediators, including insurance status, housing instability, transportation, and education.^20–22^ Although the impact of social factors on health is incontrovertible, the biological effects of these stressors may substantially mediate this relationship.^23^ Individuals living in socially vulnerable neighborhoods are exposed to chronic psychological and physiological stress, which triggers adjustments in physiological systems to maintain homeostasis.^24^ Prolonged or excessive stress can lead to maladaptive responses and biological damage.^8^ However, the physiological mechanisms linking lower socioeconomic status to an increased risk of poor surgical outcomes remain poorly understood. As such, this is the first study to characterize the impact of biological correlates of internalized stress on postoperative outcomes among patients with HPB cancer. Of note, AL increased in a stepwise manner with increasing SVI. Additionally, high AL correlated with an increased incidence of CD grade IV complications, ICU admissions, extended LOS, FTR, and 30-day mortality. Taken together, these findings underscore the elevated risk of poor surgical outcomes among patients with HPB cancer residing in socially vulnerable neighborhoods. In turn, AL can be used to target vulnerable patients in need of additional perioperative programs to mitigate their perioperative risk.

The physiologic response to stress involves a concerted effort across multiple systems to maintain internal homeostasis.^7,8^ Key mediators of this response include the production of cortisol and catecholamines.^8^ This coordinated reaction, involving the neuroendocrine system and the hypothalamic-pituitary-adrenal axis, activates the sympathetic nervous system to release epinephrine and norepinephrine.^25^ These biochemical signals prepare the body for an acute physiologic response to the stressor, triggering various cellular processes such as selective gene activation and the production of leukocytes, cytokines, and other immune mediators.^26,27^ Of note, these responses are essential for meeting the demands of immediate stress.^8^ However, prolonged activation leads to maladaptive changes in biological pathways and results in allostatic overload.^28,29^ The findings of the current study support this relationship, demonstrating a dose-response association between exposure to socioeconomic stressors and AL among patients with HPB cancer. These associations have previously been studied among individuals without cancer.^30,31^ For instance, Ribeiro et al. and Prior et al. noted that greater neighborhood socioeconomic deprivation was directly associated with increased AL.^30,32^ Similarly, Robinette et al. identified a link between symptoms of anxious arousal and elevated AL, with affective factors accounting for approximately 12% of the association between neighborhood socioeconomic status and AL.^31^

The current study builds on prior research highlighting a biological pathway that contributes to poor outcomes among patients living in socially vulnerable neighborhoods.^8,28^ Notably, high AL was associated with an increased risk of postoperative complications and FTR. At the cellular level, prolonged activation of the stress response leads to biological dysregulation across multiple systems, disrupting the healing process.^27,33^ For instance, elevated cortisol levels can impair glucose metabolism and wound healing, increasing susceptibility to infections.^33^ In addition, high AL is linked to increased inflammation, which can further complicate recovery.^27^ Although inflammation is essential for healing, excessive or dysregulated inflammatory responses can result in poor outcomes such as organ dysfunction or sepsis.^33^ In the current study, high AL was associated with an elevated risk of myocardial infarction, venous thromboembolism, surgical site infection, respiratory failure, pneumonia, and hemorrhage following surgery. These findings are consistent with previous research on lung and breast cancers.^16^ For example, Chen et al. demonstrated that higher AL was associated with postoperative complications.^16^ Furthermore, Obeng-Gyasi identified a link between high AL and increased all-cause mortality among patients with both breast cancer and non-small cell lung cancer.^12,34^ Ultimately, complex dysregulation of cardiovascular, immune, and endocrine system function makes it difficult to rescue patients from major complications and places individuals at greater risk of mortality.

The findings of the current study have several important implications to improve surgical outcomes among patients with HPB cancer.^35^ Notably, after accounting for baseline characteristics, AL was an independent predictor of postoperative complications. As such, these findings further support AL as a promising tool for preoperative risk stratification over traditional comorbidity-based risk assessment models.^36^ The clinical development of comorbid conditions represents the culmination of significant physiologic dysfuntion and maladaptive responses.^37,38^ As such, AL serves as an early evaluation of the body’s physiological stress and measures the biochemical and neuroendocrine dysfunction that occurs prior to disease presentation.^37,38^ Early identification of patients with elevated AL could inform broader perioperative interventions. Such patients may benefit from strategies that address stress biology directly, including mindfulness programs, cognitive behavioral therapy, and social support services.^39^ Integrating these approaches with nutritional optimization, metabolic control, and routine prehabilitation could blunt acute surgical stress responses and support recovery.^39^ Importantly, AL is an easily calculated measure using biomarkers such as blood pressure, glucose levels, and inflammatory markers, which are part of routine preoperative care.^12^ Consequently, AL may be integrated into clinical practice without any additional burdens of costly or invasive testing.^12^ Furthermore, using biomarkers commonly collected as part of routine preoperative work-up standardizes care across all individuals, which may provide opportunities to improve disparities in cancer care.^36^

While utilizing a large, nationally representative patient sample, the conclusions of the current study should be evaluated in consideration of its limitations in study design, patient population, outcome measures, and biases. Notably, employing a cross-sectional analysis limited the ability to determine causal relationships. Furthermore, the study included adult patients aged 18 years or older with HPB cancer, restricting its generalizability to other age groups or different medical diagnoses. Moreover, the study utilized a single measurement of AL, preventing an evaluation of its variations over time or longitudinal effects. While the Epic Cosmos database provided extensive patient- and community-level data, it lacked granular hospital-specific characteristics. Outcomes after HPB surgery are closely tied to the quality of perioperative care, with strong evidence demonstrating a volume–outcome relationship in which treatment at high-volume centers and by experienced teams is associated with lower morbidity and mortality.^40^ Because Epic Cosmos did not capture facility identifiers, hospital surgical volume, surgeon specialty, or information on access to specialized perioperative services, we were unable to assess the extent to which observed associations between AL and postoperative outcomes may reflect differences in care delivery. Future studies integrating patient-level physiologic measures such as AL with institutional characteristics are needed to clarify whether disparities in outcomes were driven by patient biology, differences in access to expert care, or a combination of both.

## Conclusion

Individuals and their health are greatly influenced by their environmental exposures, with those residing in socially vulnerable areas experiencing numerous stressors that may lead to long-term physiologic damage. The resulting disruption of biological processes increase the risk of AL, which was associated with subsequent postoperative complications and mortality. Patients from socially disadvantaged neighborhoods may require additional perioperative screening of AL and implementation of optimization strategies to mitigate disparities in HPB cancer surgical outcomes.

## Supplementary Information

Below is the link to the electronic supplementary material.

Supplementary file1 (DOCX 16 KB)

## Data Availability

The data for this study were obtained from the Epic Cosmos database. There are restrictions to the availability of these data, which is used under license for this study. Data can be accessed with permission from the Epic Systems Corporation.
