# Supplementary material for: Impact of Social Vulnerability and Allostatic Load on Postoperative Outcomes
Source: Ann Surg Oncol. 2025 Aug 20;33(2):1068–75. doi: 10.1245/s10434-025-18144-5 (PMC12765727; doi:10.1245/s10434-025-18144-5)
Supplement: Supplementary file 1 — Supplementary file1 (DOCX 16 KB) [file 10434_2025_18144_MOESM1_ESM.docx]

**Supplementary Table 1: International Classification of Diseases codes for identifying hepatopancreatobiliary cancers.**

| **Cancer type** | **ICD-10-CM** |
| --- | --- |
| Liver | C22.0, C22.1, C22.2, C22.3,  C22.4, C22.7, C22.8, C22.9 |
| Pancreas | C25.0, C25.1, C25.2, C25.3,  C25.4, C25.7, C25.8, C25.9 |
| Gallbladder and extrahepatic biliary tract | C23.0, C24.0, C24.8, C24.9 |

**Supplementary Table 2:** Risk factors associated with high allostatic load.

| **Factors** | **Odds ratio (95% CI)** |
| --- | --- |
| Age | 1.01 (1.012 – 1.017) |
| Sex |  |
| Female | Ref |
| Male | 1.70 (1.60 – 1.80) |
| Charlson comorbidity Index |  |
| ≤2 | Ref |
| >2 | 2.21 (2.02 – 2.44) |
| Race |  |
| White | Ref |
| Black | 1.53 (1.41 – 1.66) |
| Asian | 0.60 (0.50 – 0.70) |
| Other | 0.96 (0.82 – 1.11) |
| Social Vulnerability Index |  |
| Low | Ref |
| Medium | 1.11 (1.04 – 1.19) |
| High | 1.17 (1.11 – 1. 20) |
| Residential area |  |
| Metropolitan | Ref |
| Non-metropolitan | 1.09 (1.02 – 1.17) |
